# Design optimization and characterization of a dual-reporter Nipah virus pseudovirus

**DOI:** 10.52601/bpr.2025.250063

**Published:** 2026-10-31

**Authors:** Yufei Tian, Ruixin Kuang, Ruijuan Xu, Yang Qu, Ying Liao, Cuiping Song, Lei Tan, Chan Ding, Xusheng Qiu, Yingjie Sun

**Affiliations:** 1 College of Animal Science, Fujian Agriculture and Forestry University, Fujian 350002, China; 2 Shanghai Veterinary Research Institute, Chinese Academy of Agricultural Science, Shanghai 200241, China; 3 School of Agriculture and Biology, Shanghai Jiao Tong University, Shanghai 200240, China; 4 Jiangsu Co-innovation Center for Prevention and Control of Important Animal Infectious Diseases and Zoonoses, College of Veterinary Medicine, Yangzhou University, Yangzhou 225009, Jiangsu, China

**Keywords:** Nipah virus, Pseudovirus, VSV-ΔG, Dual-reporter, GFP, NanoLuciferase

## Abstract

Nipah virus (NiV), a highly pathogenic agent causing severe neurologic and respiratory disease, requires Biosafety Level 4 (BSL-4) containment. While existing pseudovirus models (*e*.*g*., based on human immunodeficiency virus (HIV) or vesicular stomatitis virus (VSV) backbones) offer alternatives, their utility is often limited by reliance on a single reporter gene. Here, we developed and optimized a robust NiV pseudovirus system utilizing a VSV-ΔG backbone co-expressing dual reporters, Green Fluorescent Protein (GFP) and NanoLuciferase (NLuc). Preliminary characterization showed that VSV-ΔG-GFP/NLuc exhibited structural integrity, achieved infection rates up to 70% in susceptible cells, and demonstrated relative stability within five freeze-thaw cycles. The study on the correlation between the expression of ephrin-B2 (EFNB2) in various cell lines and the infection efficiency of pseudovirus indicates the effectiveness of the pseudovirus. Furthermore, the platform reliably detected dose-dependent neutralization by established monoclonal antibodies targeting the fusion protein and glycoprotein of NiV, yielding trends comparable to authentic virus neutralization. Collectively, these findings establish this dual-reporter NiV pseudovirus as a safe, scalable, and high-throughput tool for antiviral screening and mechanistic studies under BSL-2 conditions. The synergistic combination of GFP (enabling visual quantification and sorting) and NLuc (providing ultra-sensitive, broad dynamic range quantification) enhances both versatility and data reliability. This study not only provides a critical new tool for NiV research but also serves as a valuable reference for developing pseudovirus platforms targeting other enveloped viruses.

## INTRODUCTION

Nipah virus (NiV), a representative zoonotic pathogen of the genus Henipavirus (HNV) within the family *Paramyxoviridae*, poses a serious threat to global public health (Spengler *et al.* 2025; Tan *et al.* 2024). NiV infection can cause acute encephalitis and respiratory failure, with a fatality rate as high as 40%−75%, and survivors often suffer permanent neurological deficits (Sharma *et al.* 2024). Due to its high lethality, lack of effective vaccines or specific antiviral drugs, and potential bioterrorism risk, NiV is listed on the World Health Organization (WHO)’s R&D Blueprint priority pathogen list (Moore *et al.* 2024). The molecular mechanisms driving NiV entry depend on its two envelope glycoproteins: the glycoprotein (G protein) mediates binding to host cell receptors, and the fusion protein (F protein) catalyzes membrane fusion (Spengler *et al.* 2025). However, research into these mechanisms and the development of countermeasures face a significant obstacle: NiV requires Biosafety Level 4 (BSL-4) containment due to its extreme lethality and potential for aerosol transmission (Spengler *et al.* 2025; van den Hurk *et al.* 2025). With fewer than 50 operational BSL-4 facilities globally, their high costs, lengthy regulatory approval cycles, and stringent biocontainment restrictions severely hinder progress in neutralizing antibody assessment, vaccine development, and antiviral drug screening. In this context, the development of pseudovirus surrogate technologies that can be safely manipulated in BSL-2 laboratories emerges as a strategic solution to accelerate NiV research.

Pseudovirus systems significantly reduce biosafety risks by incorporating the envelope glycoproteins of highly pathogenic viruses into replication-deficient viral vectors (like Moloney murine leukemia virus (MuLV), human immunodeficiency virus (HIV), or vesicular stomatitis virus (VSV) backbones) (Li *et al.* 2018; Xiang *et al.* 2022). This allows functional mimicry of the natural viral entry mechanism while completely eliminating autonomous replication capability, enabling related research to be conducted in BSL-2 laboratories. Although such platforms are widely used in enveloped virus research due to their flexible reporter gene integration technology, the current NiV pseudovirus systems are constrained by inherent limitations of single-reporter genes: fluorescent proteins (*e*.*g*., Green Fluorescent Protein (GFP)/Red Fluorescent Protein (RFP)/ZsGreen(ZsG)) enable real-time tracking of infection dynamics and support microscopic imaging, but their low sensitivity and narrow quantitative range make them inadequate for capturing weak infection events (Jain *et al.* 2023; Porotto *et al.* 2011; Wang *et al.* 2024a); traditional luminescent enzymes (*e*.*g*., Firefly/Renilla Luciferase (FLuc/RLuc)) offer high sensitivity, but their rapidly decaying signals prevent spatial localization of infection foci (Nie *et al.* 2019; Wang *et al.* 2024b); secreted tags (*e*.*g*., secreted alkaline phosphatase (SEAP)) are suitable for high-throughput screening, yet their delayed response misses critical early infection processes (Varghese *et al.* 2025). These single-reporter systems fail to simultaneously meet the requirements for high-sensitivity quantification and multidimensional dynamic analysis. Furthermore, they are prone to interference from false signals, compromising data reliability (Thimmiraju *et al.* 2024). Consequently, developing novel platforms integrating multiple reporter genes has become a critical breakthrough point for overcoming current technical bottlenecks, deepening the understanding of viral entry mechanisms, and accelerating antiviral drug development.

The VSV-ΔG backbone, owing to its high packaging efficiency (>10^7^ TU/mL), broad cellular tropism, and strictly single-cycle infection properties, has become the preferred platform for constructing NiV pseudoviruses (Li *et al.* 2018; Xiang *et al.* 2022). This system replaces the VSV-G gene with exogenous NiV-G/F expression and integrates reporter genes to achieve virus assembly. To overcome the limitations of single-reporter systems, this study pioneered a NiV dual-reporter pseudovirus based on the VSV-ΔG backbone, synergistically integrating two high-performance reporter elements: NanoLuciferase (NLuc) — with an ultra-small molecular weight of 19 kDa (one-third that of traditional luciferases), it minimizes steric hindrance while catalyzing the furimazine substrate to produce a 100-fold enhanced luminescent signal. Capable of detecting infection rates as low as 0.1% and featuring a signal half-life >2 h, it supports sustained kinetic monitoring (Cruz-Cardenas *et al.* 2022; England *et al.* 2016). Classic GFP — leveraging its rapid maturation (<30 min) and optimal excitation wavelength of 488 nm, it enables live-cell imaging and flow cytometric sorting (Yang *et al.* 2021). In this study, we developed and optimized an efficient packaging system for the VSV-ΔG-GFP/NLuc dual-reporter pseudovirus, evaluated the stability and functionality of this dual-reporter pseudovirus, and further explored its value in studying viral entry mechanisms and assessing neutralizing antibodies. This platform not only provides a core tool for evaluating vaccine immunogenicity, discovering broadly neutralizing antibodies, and screening small-molecule inhibitors, but can also be extended to research on other viruses of the genus HNV and highly pathogenic enveloped viruses.

## RESULTS

### Production and optimized protocol for NiV pseudoviruses

The general scheme of experiments followed to produce and detect the NiV pseudovirions is shown. Briefly, cells were transfected with NiV-F and NiV-G plasmids, infected 24 h later with VSV-ΔG-GFP/NLuc to generate pseudoviruses, and supernatants were harvested at 48 h and stored; packaging efficiency was read out by GFP flow cytometry and NLuc luminescence. Packaging efficiency was evaluated in parallel by flow cytometric detection of GFP-positive cells and by RLUs from the NLuc assay (Fig. 1A). Initial pseudovirus packaging using pcDNA3.1-based envelope plasmids encoding NiV-M F and G glycoproteins yielded suboptimal titers, prompting a series of optimization steps. Replacing the Malaysian strain with the Bangladeshi strain significantly reduced the titer (*P* < 0.001), so the NiV-M pseudovirus was selected for follow-up study (Fig. 1B). Previous studies have shown that truncation of F and G proteins by deleting 138 amino acids from the C-terminus of NiV-M F and 99 amino acids from the C-terminus of NiV-M G will improve the packaging efficiency of pseudovirus (Larsen *et al.* 2025), but this phenomenon was not observed in our study (Fig. 1C). In contrast, switching the expression vector from pcDNA3.1 to pCAGGS significantly improved packaging efficiency, yielding 2.2-fold higher transduction by flow cytometry (*P* < 0.001) and 2.3-fold higher luminescence by NLuc assay (*P* < 0.0001) (Fig. 1D).

**Figure 1:**
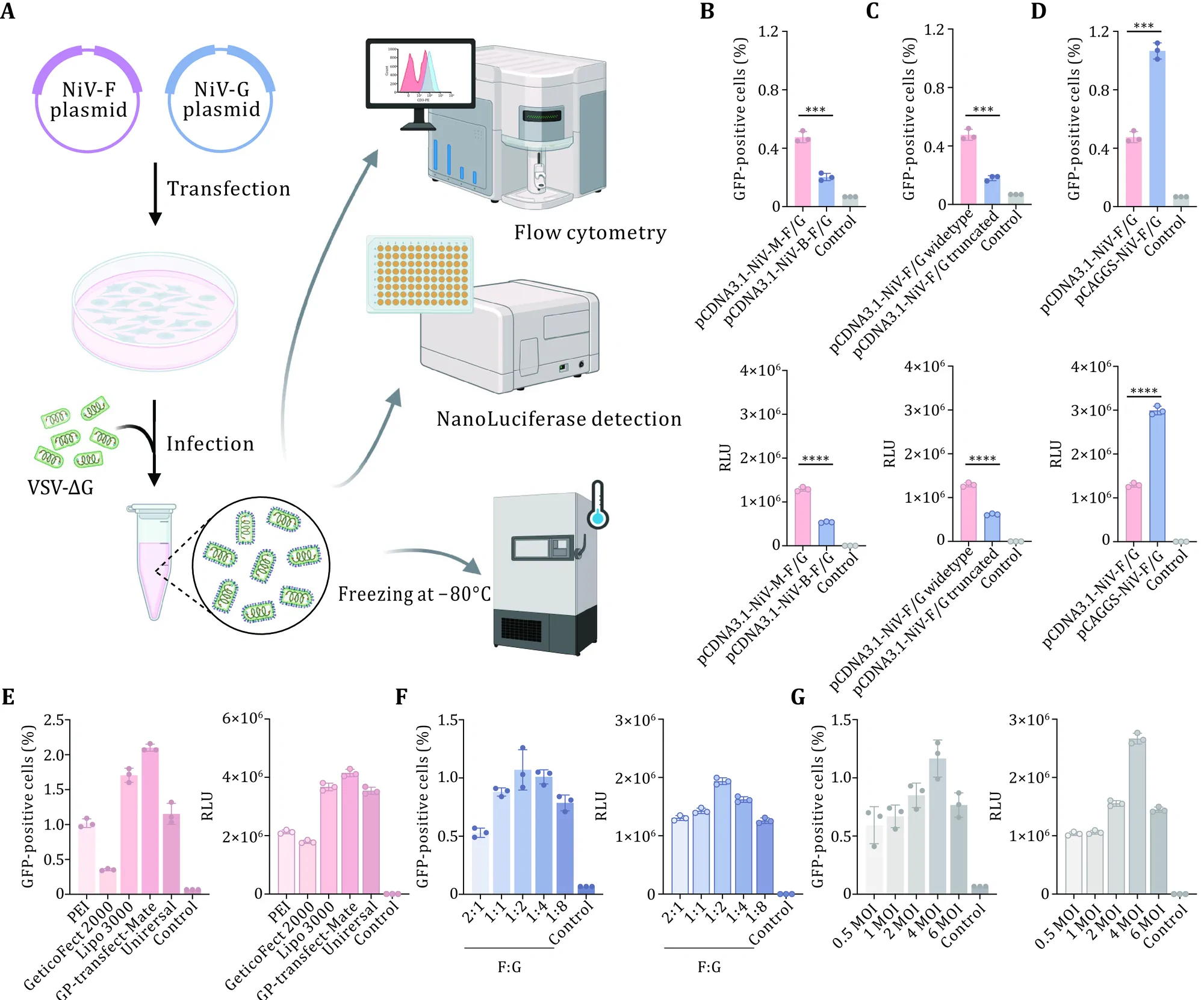
Preparation and optimization of NiV pseudovirus. **A** Scheme of experiments for generation and detection of NiV pseudovirions. **B**−**D** The packaging conditions of pseudovirus were optimized by changing the envelope plasmid: different F and G expression plasmids: pcDNA3.1-NiV-M-F/G and pcDNA3.1-NiV-B-F/G (**B**), pcDNA3.1-NiV-M-F/G and pcDNA3.1-NiV-M-F_ΔC138_/G_ΔC99_ (**C**), and pcDNA3.1-NiV-M-F/G and pCAGGS-NiV-M-F/G (**D**) were used for NiV pseudovirus packaging. Untransfected cells served as negative controls. The harvested virus was used to infect HEK293T target cells, and the quantity of NiV pseudovirus in infected cells was determined 24 h after infection. Flow cytometry was used to detect the level of GFP^+^ in HEK293T cells (above), and a NanoLuciferase (NLuc) assay was used to detect the level of NLuc activity in HEK293T cells (below). **E**−**G** The packaging conditions of pseudovirus were optimized by changing three parameters: using pCAGGS-NiV-M-F/G expression plasmids in different transfection reagents (PEI, GeticoFect 2000, Lipofectamine 3000, GP-transfect-Mate, Hieff Trans Universal) (**E**), F∶G plasmid ratio (2∶1, 1∶1, 1∶2, 1∶4, 1∶8) (**F**), and MOI (0.5, 1, 2, 4, 6) (**G**). Untransfected cells served as negative controls. The harvested virus was used to infect HEK293T target cells, and the quantity of NiV pseudovirus in infected cells was determined 24 h after infection. Flow cytometry was used to detect the level of GFP^+^ in HEK293T cells (left), and an NLuc assay was used to detect the level of NLuc activity in HEK293T cells (right). Experiments were done in duplicates, and data were presented as mean ± SD. Un-paired Student *t*-test was used for statistical analysis comparing the two groups, *P < 0.05, **P < 0.01, ***P < 0.001, ****P < 0.0001, and ns, not significant

Further optimization of production parameters revealed that, compared with other transfection reagents, GP-transfect-Mate yielded the highest titers for NiV pseudovirus packaging (Fig. 1E). Systematic variation of envelope plasmid ratios showed that increasing the pCAGGS-G proportion enhanced titers, with peak production at a F:G ratio of 1:2, yielding a 1.2-fold increase by flow cytometry and a 1.4-fold increase by NLuc relative to 1:1. (Fig. 1F). Additionally, pseudovirus production increased with MOI, peaking at MOI 4, with a 1.7-fold increase by flow cytometry and a 2.5-fold increase by NLuc relative to MOI 1 (Fig. 1G). Concordant trends in both GFP^+^ cell frequency and NLuc luminescence validated these patterns. Collectively, these data define the optimal production conditions for high-titer NiV pseudovirus: pCAGGS-F/G (NiV-M wild type), F:G ratio of 1:2, and MOI 4.

### Characterization of NiV pseudovirus

Following packaging and concentration using the described methodology, the pseudovirus demonstrated significantly enhanced infection efficiency. With further concentration, flow cytometric analysis revealed that 71% of cells infected with the concentrated pseudovirus were GFP^+^, substantially exceeding the baseline observed in uninfected controls (Fig. 2A), a finding corroborated by direct visualization under fluorescence microscopy (Fig. 2B). Structural characterization via negative-stain electron microscopy confirmed the correct morphology of individual NiV pseudovirus particles. Consistent with their VSV-based backbone, the majority of particles exhibited the characteristic bullet-shaped structure, measuring approximately 170 nm in length and 70 nm in width (Fig. 2C). Stability testing, quantified by NLuc activity, indicated a gradual decline in pseudovirus titer over the first five freeze-thaw cycles (average reduction of ~2 × 10^5^ RLU per cycle). However, a marked decrease in titer (reduction of ~1 × 10^6^ RLU) was observed following the sixth cycle (Fig. 2D). Collectively, these results demonstrate the successful production and concentration of morphologically authentic pseudoviruses, achieving high functional titers, while highlighting the necessity for aliquot storage to mitigate titer loss associated with repeated freeze-thaw cycles.

**Figure 2:**
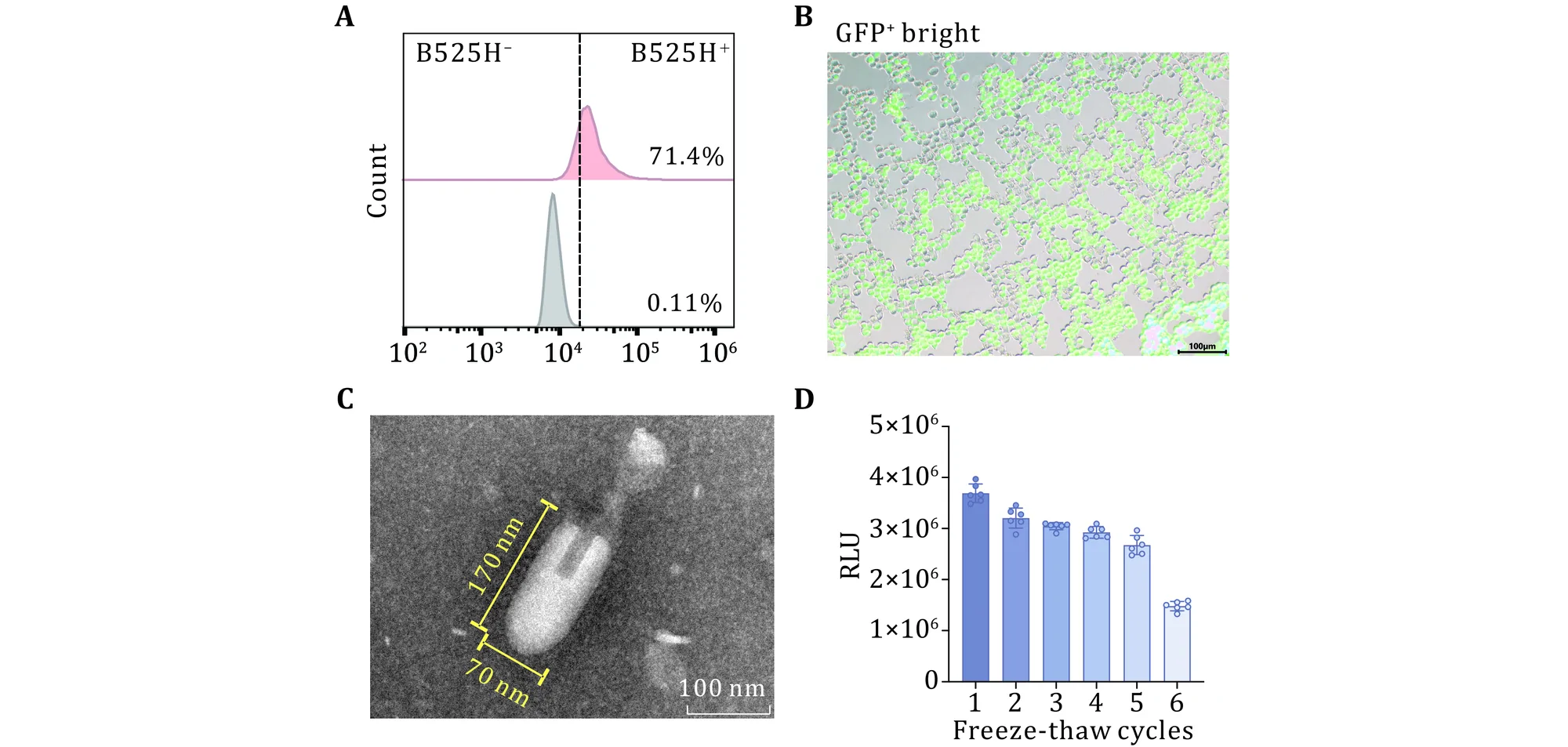
Characterization of NiV pseudovirus. **A**,**B** The harvested virus was concentrated by centrifugation at 4000 r/min for 10 min, then used to infect HEK293T target cells, and observed and detected 24 h after infection. Flow cytometry was used to detect the GFP^+^ level in HEK293T cells (**A**). GFP^+^ bright cells were observed by fluorescence microscope (Scale bar, 100 μm) (**B**). **C** The NiV pseudovirus was concentrated and negatively stained, and its morphology was observed under a transmission electron microscope operated at 80 kV (Magnification, 150,000×; Scale bar, 100 nm). **D** The pseudovirus was divided into equal parts, and freeze-thaw cycles (−80°C to 37°C) were carried out for 1−6 times, respectively. The treated pseudovirus was then used to infect HEK293T target cells, and NLuc activity was detected 24 h after infection to evaluate the stability of the pseudovirus. Experiments were done in duplicates, and data were presented as mean ± SD

### Correlation analysis of NiV pseudovirus cell tropism and EFNB2 receptor expression

EFNB2 is a classical NiV receptor (Bonaparte *et al.* 2005). To verify the pseudovirus’s effectiveness, we evaluated the consistency between EFNB2 expression across various cell lines correlates with infection efficiency. qPCR analysis revealed substantial heterogeneity in EFNB2 transcripts across cell lines. U251 and Huh-7 showed the highest expression (approximately 3−4-fold higher than HEK293T). HEK293T, A549, and BHK-21 exhibited moderate levels. Low expression was observed in human cells (AC16, H1299, HeLa, HCT116) and in cross-species lines, including Vero E6 (African green monkey), CHO-K1 (hamster), PK15 (porcine), MDCK (canine), and DF-1 (avian), all less than half of that in HEK293T (Fig. 3A).

**Figure 3:**
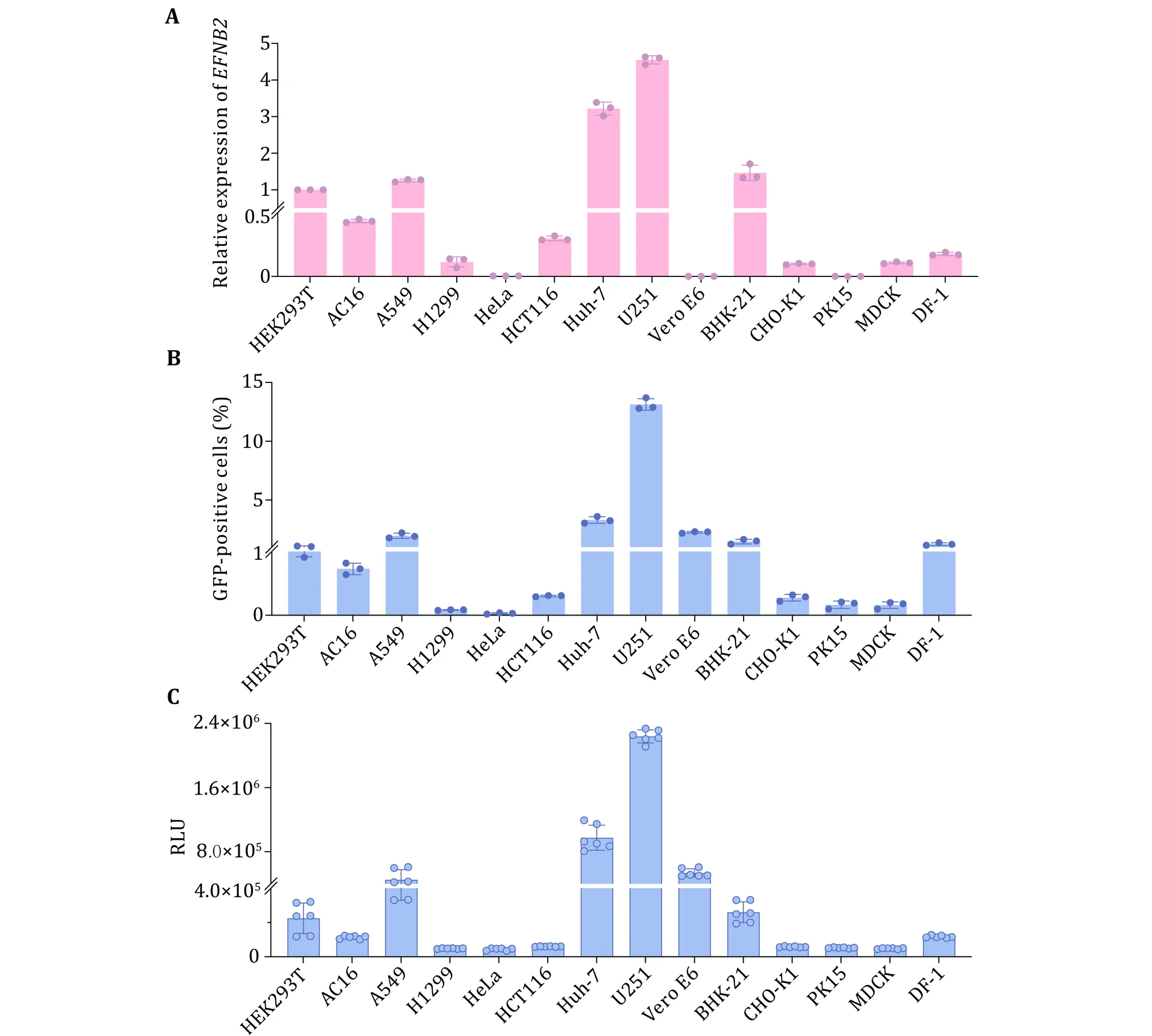
Expression of *EFNB2* and tropism determination of NiV pseudovirus in 14 cell lines. **A** RNA samples from 14 kinds of cells (HEK293T, AC16, A549, H1299, HeLa, HCT116, Huh-7, U251, Vero E6, BHK-21, CHO-K1, PK15, MDCK, and DF-1 cells) were collected and reverse transcribed, and then the expression level of *EFNB2* at the transcriptional level was detected by qPCR. Relative mRNA expression levels for each sample were normalized to *EFNB2* mRNA expression in HEK293T cells. **B**,**C** The NiV pseudovirus was infected with 14 different target cells for 24 h. Flow cytometry was used to detect GFP^+^ level (**B**), and the NLuc assay was used to detect NLuc activity level (**C**). Experiments were done in duplicates, and data were presented as mean ± SD

Viral susceptibility assays via flow cytometry (GFP^+^ cells) and the NLuc activity assay demonstrated concordant tropism patterns: NiV pseudovirus exhibited maximal infectivity in U251 cells (13.0-fold by flow cytometry; 9.9-fold by NLuc assay relative to HEK293T controls), moderate susceptibility in HEK293T, A549, Huh-7 (human), Vero E6 (African green monkey), and BHK-21 (hamster) cells (1-5-fold by flow cytometry and NLuc assay relative to HEK293T controls), and minimal entry in H1299, HeLa, HCT116 (human), CHO-K1 (hamster), PK15 (porcine), MDCK (canine), and DF-1 (avian) cells (Figs. 3B and 3C). Collectively, viral entry efficiency correlated positively with EFNB2 abundance in most cell types, though significant deviations from this correlation were observed in H1299 (high receptor protein/low infection) and Vero E6 (low receptor/moderate infection).

### Functional validation of EFNB2-dependent viral entry

To further validate the pseudovirus’s robustness, we assessed infection efficiency in target cells after EFNB2 overexpression and knockdown. Previous studies have shown that HEK293T cells can be used to carry out pseudovirus invasion experiments, while CHO-K1 cells and DF-1 cells prove insensitive to NiV pseudovirus infection — a finding that has been verified in our current study (Cui *et al.* 2024). In susceptible HEK293T cells, EFNB2 knockdown using two independent siRNAs significantly reduced receptor protein levels, as confirmed by Western blot analysis. This reduction correlated with markedly diminished viral entry, evidenced by decreased GFP^+^ cell frequency (1.6-fold for si-EFNB2-1, *P* < 0.001, and 2.9-fold for si-EFNB2-2, *P* < 0.0001) and reduced NLuc activity (2.8-fold for si-EFNB2-1, *P* < 0.0001, and 4.8-fold for si-EFNB2-2, *P* < 0.0001) compared to scramble controls (Fig. 4A). Conversely, EFNB2 overexpression in non-susceptible CHO-K1 and DF-1 cells restored viral entry competence, with successful receptor expression verified by Western blot. Functional assays confirmed significantly enhanced pseudovirus infection in CHO-K1 by flow cytometry (4.2-fold, *P* < 0.05) and by NLuc assay (2.1-fold, *P* < 0.0001), or in DF-1 by flow cytometry (2.1-fold, *P* < 0.0001) and by NLuc assay (1.5-fold, *P* < 0.0001) (Fig. 4B). These experiments further confirm the validity of our pseudovirus, showing that its entry is EFNB2-dependent and that modulating EFNB2 expression yields consistent infection changes across multiple cell lines.

**Figure 4:**
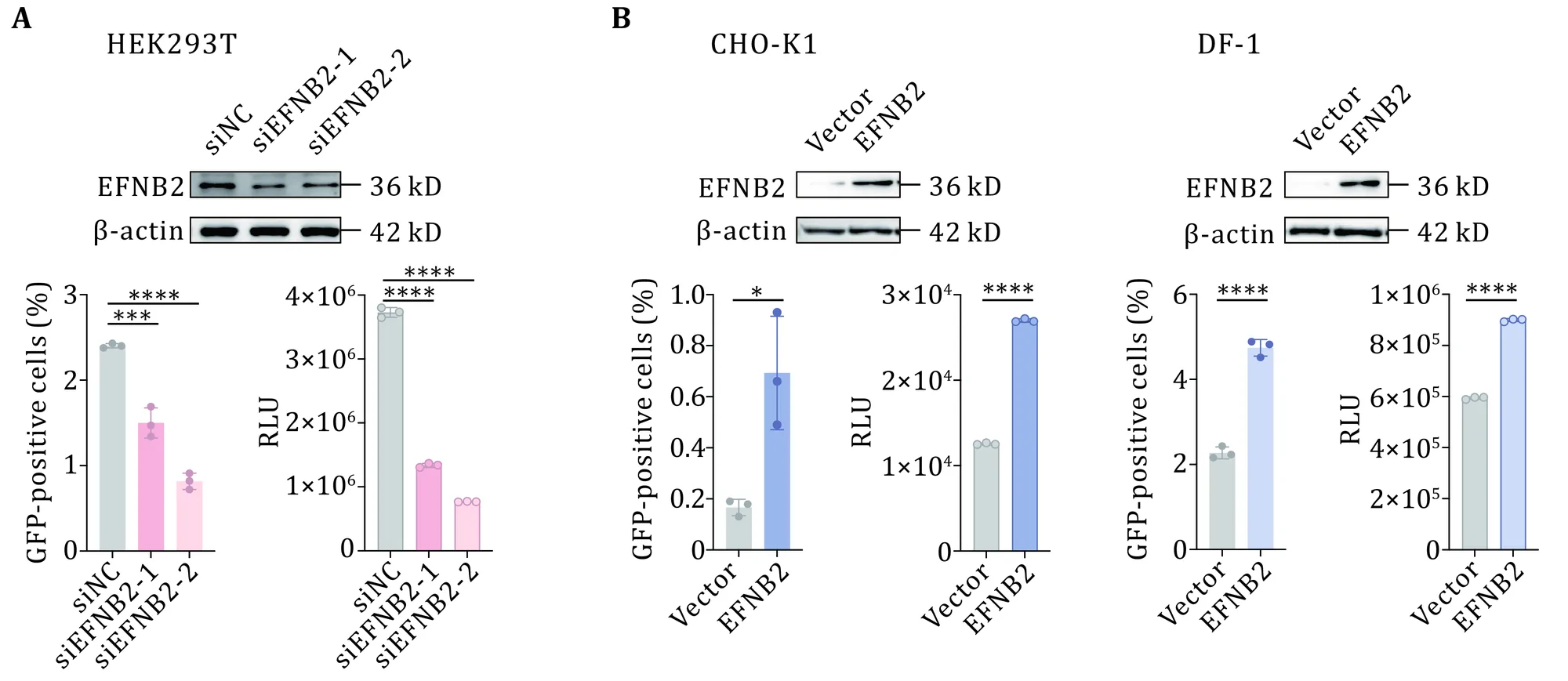
Functional validation of EFNB2-dependent viral entry. **A** HEK293T cells were transfected with siNC or siEFNB2-1/2, and infected with NiV pseudovirus 48 h later. **B** CHO-K1 and DF-1 cells were transfected with vector or EFNB2, and infected with NiV pseudovirus 24 h later. The protein levels of EFNB2 and β-actin were analyzed by Western blot. Flow cytometry was used to detect the level of GFP^+^ in HEK293T cells (left), and an NLuc assay was used to detect the level of NLuc activity in HEK293T cells (right). Experiments were done in duplicates, and data were presented as mean ± SD. Un-paired Student *t*-test was used for statistical analysis comparing the two groups, *P < 0.05, **P < 0.01, ***P < 0.001, ****P < 0.0001, and ns, not significant

### Functional validation of NiV pseudoviruses through antibody neutralization

The functional authenticity of pseudotyped virions was assessed using monoclonal antibodies (mAbs) against NiV glycoproteins in standardized neutralization assays. All tested monoclonal antibodies — 5B3, 1F5, 1H1 (anti-F) and m102.4 (anti-G) — displayed canonical four-parameter logistic inhibition curves when neutralization percentage was plotted against the log-transformed antibody concentrations (Fig. 5). Within the 15%−95% neutralization range, the luciferase readout showed a linear relationship between antibody dilution factor and inhibition rate. The half-maximal inhibitory concentrations (*IC*_50_) were 0.07 μg/mL for m102.4, 0.33 μg/mL for 5B3, 0.05 μg/mL for 1F5, and 1.67 μg/mL for 1H1, demonstrating differential neutralization potency corresponding to their respective glycoprotein targets. These results confirm the structural and functional integrity of the pseudovirus system for modeling antibody-mediated neutralization.

**Figure 5:**
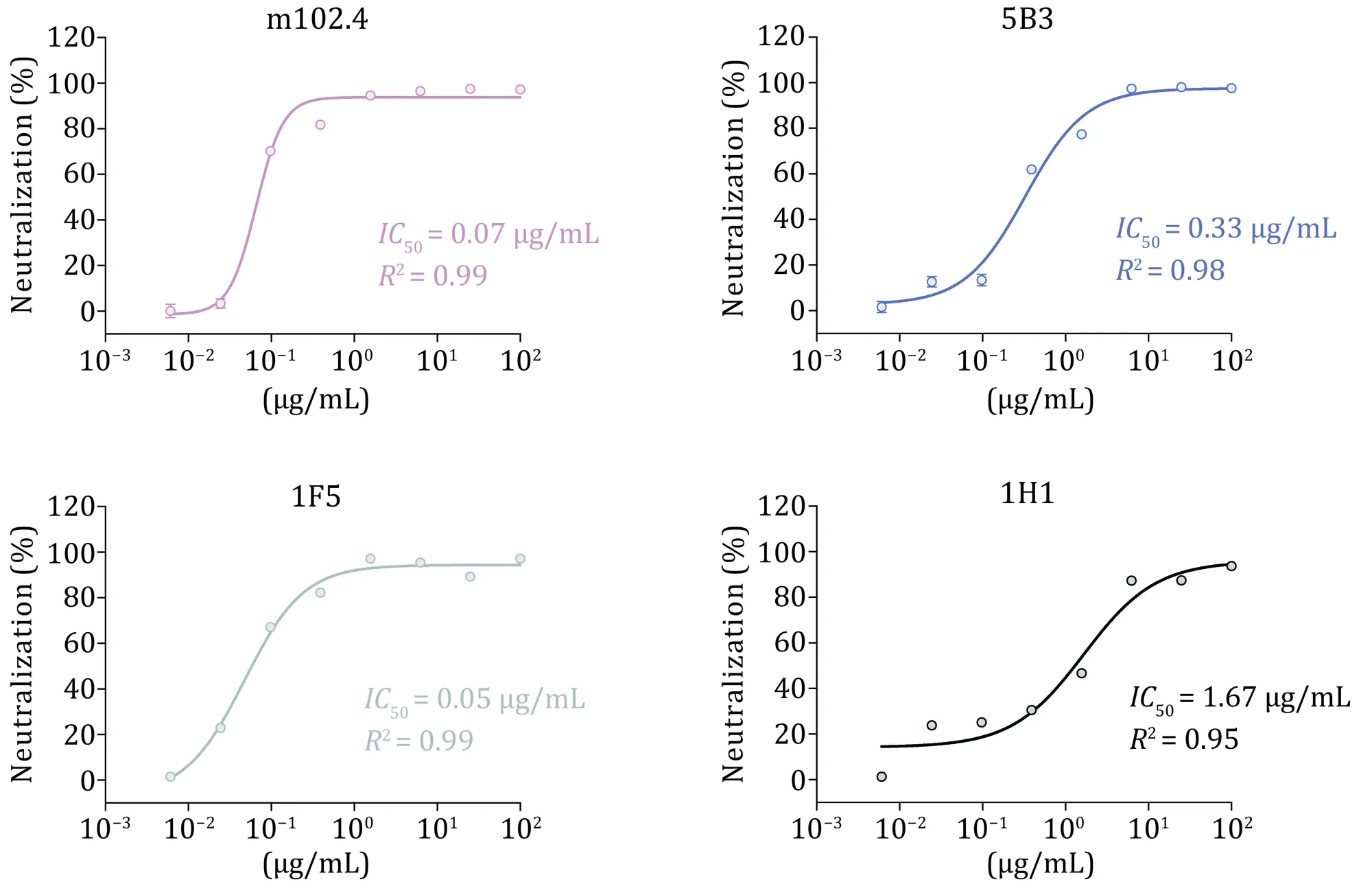
Validation of neutralization ability of NiV pseudovirus. The previously reported monoclonal antibodies m102.4, 5B3, 1F5, and 1H1 were continuously diluted four times with the initial concentration of 100 μg/mL, then incubated with pseudovirus at a 1:1 volume ratio for 1 h at 37°C. The antibody-pseudovirus mixtures were applied to HEK293 cells. After 24 h, an NLuc assay was used to detect the level of NLuc activity. Dose-response curves were fitted using a four-parameter logistic model, and the corresponding *IC*_50_ and *R*^2^ values were listed. Experiments were done in duplicates, and data were presented as mean ± SD

## DISCUSSION

The absence of approved vaccines or therapeutics for NiV, a high-mortality pathogen requiring BSL-4 containment, underscores the critical need for accessible research tools. While single-reporter pseudoviruses have served as BSL-2 alternatives, they cannot give consideration to both spatial resolution and quantitative sensitivity. In this study, we developed a double reporter gene (GFP/NLuc)-expressing NiV pseudovirus platform, and then completed the system optimization and comprehensive characterization, including cell tropism, receptor dependence and neutralizing activity. This platform provides a powerful tool for accelerating the discovery of entry inhibitors, therapeutic antibodies and candidate vaccines against NiV.

Previous studies have utilized MuLV, HIV, and VSV as backbones for pseudovirus packaging (Bae *et al.* 2019; Nie *et al.* 2019; Varghese *et al.* 2025). Compared to MuLV, HIV-based lentiviral vectors typically possess a larger packaging capacity and enhanced ability to infect non-dividing cells (Dull *et al.* 1998; Naldini *et al.* 1996). In contrast, the VSV backbone exhibits a series of superior characteristics that make it more suitable for both *in vitro* and *in vivo* studies, including: the unique capability for efficient incorporation of heterologous envelope proteins (Takada *et al.* 1997); high packaging efficiency and the safety of single-round infection (Schnell *et al.* 1996); broad host/envelope protein compatibility and operational simplicity, making it an ideal platform for high-throughput assays (Whitt 2010). Based on these advantages, we selected VSV as the pseudovirus packaging backbone to ensure its broad applicability in research such as viral entry mechanisms and neutralizing antibody detection. Reporters function as molecular probes by transforming molecular events into perceptible, quantifiable, and traceable signals, making their selection a critical step in pseudovirus construction (Chalfie *et al.* 1994; Contag *et al.* 1997; Dyer *et al.* 2000). Currently, commonly used reporter genes for NiV pseudoviruses fall into two main categories: fluorescent proteins: typically carried by the VSV backbone, utilize intrinsic fluorescence to enable rapid, intuitive assessment of serum/antibody neutralizing activity (Chen *et al.* 2024), vaccine immunogenicity (Chattopadhyay and Rose 2011), and receptor-mediated inhibition of viral entry (Porotto *et al.* 2011) by quantifying post-infection positive cells or fluorescence intensity; and enzymatic reporters: which require substrate reactions to generate signals, including SEAP for direct supernatant assay in high-throughput automation (*e*.*g*., PVNT), albeit with limited sensitivity (Varghese *et al.* 2025); β-Galactosidase (LacZ) for intuitive qualitative/retrospective analysis via X-Gal staining, though hindered for large-scale use by cumbersome procedures and subjectivity (Bae *et al.* 2019); and luciferase (luc), widely used with HIV or VSV backbones and requiring cell lysis *in vitro* or substrate injection *in vivo*, offering high sensitivity and broad dynamic range for evaluating antibody/vaccine neutralization and protective efficacy (Nie *et al.* 2019; Xu *et al.* 2012b), despite its transient signal limiting the detection window. Based on analysis of existing NiV pseudovirus systems, we engineered a synergistic dual-reporter system (GFP-NLuc), providing key advantages: multidimensional data complementarity, where NLuc delivers real-time quantitative data while GFP reveals infection spatial distribution and cellular tropism; and enhanced reliability through cross-validation, as independent detection of both reporters identifies non-specific interference (*e*.*g*., compound autofluorescence), significantly reducing false-positive rates. Although not the first dual-reporter system, compared to lentiviral vectors carrying eGFP and FLuc, the NLuc component offers ultra-high sensitivity, rapid reaction kinetics, and sustained luminescence, substantially improving detection convenience and efficiency (Xu *et al.* 2012b).

Following the initial establishment of the pseudovirus packaging protocol, systematic optimization efforts were undertaken. Notably, previous studies found that truncating the cytoplasmic tail domain reduces steric hindrance or conformational interference during viral assembly, thereby enhancing the incorporation efficiency of envelope proteins and ultimately increasing pseudovirus packaging titers (Larsen *et al.* 2025; Witting *et al.* 2013). However, no such effect was observed in the present study. This discrepancy may stem from a mismatch between the expression vector and viral backbone used, which failed to significantly increase the actual protein expression levels. A significant breakthrough was achieved, however, by switching to the pCAGGS vector. Leveraging its potent hybrid CAG promoter for superior transcriptional activity, this vector drives highly efficient glycoprotein expression (Mizushima and Nagata 1990). Concordant results from both flow cytometry and NLuc assays validated the stability of the dual-reporter system. In addition, stability assessments revealed a critical limitation: freeze-thaw cycles caused significant titer loss, with precipitous declines occurring after more than five cycles. This finding provides direct guidance for experimental practice — recommending the use of single-use aliquots to ensure data reliability — and defines a clear direction for translational applications, establishing a ≤5-cycle tolerance threshold for kit development and shipping logistics. To sum up, after optimization and verification, we have successfully developed a NiV pseudovirus platform with dual-reporter genes, which provides a reliable and multifunctional tool for the NiV research from basic construction to downstream application.

The NiV pseudovirus demonstrated broad cellular tropism across diverse animal cell lines, exhibiting varying degrees of infectivity, a characteristic feature of NiV rather than VSV. The observed infection efficiency differences directly correlated with cell-specific properties: Low-susceptibility cell lines (*e*.*g*., CHO-K1, HeLa, and H1299) likely resulted from receptor deficiency or intrinsic immune activation, while others were caused by non-natural hosts (*e*.*g*., MDCK and DF-1) or non-primary target tissues (*e*.*g*., AC16 and HCT116); high-susceptibility cells (*e*.*g*., Huh-7, Vero E6, BHK-21, and HEK293T) showed infection rates consistent with classical studies and have been extensively used in mechanistic research (Loomis *et al.* 2020; Nie *et al.* 2019). Notably, the glioma cell line U251 exhibited exceptionally high infection rates, surpassing conventionally used Huh-7 cells. Since EFNB3 is predominantly expressed in the central nervous system and neural tissues (Negrete *et al.* 2007). We therefore hypothesize that, in addition to elevated EFNB2, EFNB3 contributes to the increased infectivity observed in this glial context, aligning with prior reports of EFNB family members mediating viral entry in neural cells (Xu *et al.* 2012a). The previously unreported phenomenon aligns with NiV’s neurotropic pathogenesis targeting the central nervous system, suggesting a potential role for astrocytes in encephalitis development (Dawes and Freiberg 2019). This finding provides new perspectives for constructing neurospecific infection models and developing targeted therapies. Receptor mechanism studies confirmed that EFNB2 expression levels significantly correlate with viral entry efficiency, which was validated across 14 cell lines and confirmed by genetic manipulation, demonstrating the system’s fidelity in recapitulating authentic NiV entry traits. Critical exception cases further revealed cell-specific regulatory mechanisms: the paradox of high EFNB2 expression yet low infectivity in H1299 cells suggests post-translational modifications or endocytic barriers, while high infectivity and extremely low EFNB2 expression in Vero E6 cells indicate the existence of alternative receptor pathways. These observations highlight the intricate host-factor regulation of viral entry. Future studies should investigate the functional receptor complex in H1299 cells and elucidate molecular EFNB2-independent mechanisms in Vero E6 to refine our understanding of NiV tropism pathways.

The result confirmed that the constructed pseudovirus reliably and sensitively detects neutralizing antibody activity targeting the NiV-F/G glycoproteins. The measured IC_5__0_ values were comparable to those reported for authentic virus in the literature, demonstrating the sensitivity required to discriminate antibody efficacy (Dang *et al.* 2019, 2021; Wang *et al.* 2024b). While this platform serves as an ideal tool for evaluating therapeutic antibodies and vaccine-elicited neutralizing responses, its inherent limitations must be acknowledged: as a pseudovirus system, it solely mimics the viral entry process into host cells (including the attachment and fusion steps) and cannot recapitulate the complete viral life cycle (such as viral replication, assembly, and release) (Crawford *et al.* 2020). Furthermore, despite the high conservation of F/G glycoproteins across strains, experimental results obtained using the glycoproteins from this specific strain may not be directly extrapolated to other strains. Concurrently, co-expression of the NLuc reporter gene may partially suppress the fluorescence intensity of GFP (Taoka *et al.* 2021). This phenomenon may stem from competitive transcriptional/translational resource allocation in our dual-tagged NiV pseudovirus system (GFP/NLuc), where the high-expression NLuc may potentially compete for translational resources and attenuate GFP expression. To address this limitation, we adopted two complementary strategies: (1) further concentrating the pseudovirus to substantially increase the GFP-positive rate (Fig. 2A), and (2) prioritizing NLuc as the primary quantitative metric given its superior dynamic range while extending the post-infection incubation period before flow cytometry to enhance mature GFP accumulation. In the future, this versatile platform can support several key applications, including screening small-molecule inhibitors targeting viral entry, evaluating neutralizing responses induced by diverse vaccine strategies, and developing high-throughput neutralization assays. Additionally, the NLuc reporter gene exhibits exceptional stability, laying the groundwork for the platform’s potential application *in vivo* studies.

In summary, we have constructed and validated a fully functional dual-reporter (GFP/NLuc) NiV pseudovirus platform. This BSL-2-compatible platform synergistically integrates two key strengths: single-cell visualization via flow cytometry, enabling direct quantification of infection rates and cellular tropism analysis; and ultrasensitive quantification based on NLuc, providing a >4-log dynamic range for precise measurement of viral entry efficiency and neutralization efficacy. This powerful, safe, and efficient technological tool provides a robust foundation for in-depth studies of NiV entry mechanisms, receptor utilization, antibody neutralization, and antiviral drug development, significantly advancing NiV research and control efforts.

## MATERIALS AND METHODS

### Cells

HEK293, HEK293T, AC16, A549, HeLa, HCT116, Huh-7, U251, BHK-21, PK15, MDCK, and DF-1 cells were purchased from the Cell Bank of the Shanghai Institute of Biochemistry and Cell Biology, Chinese Academy of Science (http://www.cellbank.org.cn), and maintained in Dulbecco’s Modified Eagle Medium (DMEM, Gibco, USA) supplemented with 10% fetal bovine serum (FBS) (Gibco, USA) and 1× penicillin-streptomycin (Beyotime, China). CHO-K1 cells were maintained in the same medium supplemented with 10 mmol/L HEPES (Beyotime, China), 1 mmol/L sodium pyruvate (Beyotime, China), and 1× non-essential amino acids (Beyotime, China). H1299 and Vero E6 cells were maintained in RPMI Medium 1640 (Gibco, USA) and Minimum Essential Medium (MEM, Gibco, USA), respectively, supplemented with 10% FBS and 1× penicillin-streptomycin. All cells were cultured in a 5% CO_2_ environment at 37°C.

### Construction of the NiV envelope-expressing plasmids

For pseudovirus construction, the F and G genes from the Malaysian strain (NiV-M; GenBank: AF212302) and the Bangladesh strain (NiV-B; GenBank: AY988601) were cloned into the mammalian expression vectors pcDNA3.1 to generate NiV envelope-expressing plasmids. pCAGGS-NiV-M-F and pCAGGS-NiV-M-G were obtained from Chang Li (Changchun Veterinary Research Institute). Truncated variants were produced by deleting 138 amino acids from the C-terminus of NiV-M F and 99 amino acids from the C-terminus of NiV-M G, yielding pcDNA3.1-F_ΔC138_ and pcDNA3.1-G_ΔC99_, respectively. The primer sequences for plasmid construction are listed in Table 1.

**Table 1:** Primers used for qPCR

Primers	Application	Sequence (5’-3’)
EFNB2-L	For HEK293T, AC16, A549, H1299, HeLa, HCT116, Huh-7, U251, and Vero E6 cells	TAAAGATCCAACAAGACGTCCA
EFNB2-R		CGTGATGATGATGACGATGAAG
β-actin-L		TGTGGCCGAGGACTTTGATT
β-actin-R		CCTGTGTGGACTTGGGAGAG
EFNB2-L	For BHK-21 and CHO-K1 cells	CTGGGTTCCGAAGTGGCCTT
EFNB2-R		CCTGCGGTACTTGAGCAGCA
β-actin-L		CATTGCTGACAGGATGCAGAAGG
β-actin-R		TGCTGGAAGGTGGACAGTGAGG
EFNB2-L	For PK15 cells	TCCAGCACAGACGGCAACAG
EFNB2-R		GTCTCCGCCGGTACTTGAGC
β-actin-L		GCGCAAGTACTCCGTGTGGA
β-actin-R		GCATTTGCGGTGGACGATGG
EFNB2-L	For MDCK cells	GCCGGACATTCCGGGAACAA
EFNB2-R		GTCTCCGCCGGTACTTGAGC
β-actin-L		CCCAAGGCCAACCGTGAGAA
β-actin-R		CCGGAGTCCATCACGATGCC
EFNB2-L	For DF-1 cells	ACGCAGTGGCAACAACAATGG
EFNB2-R		TGCTGGACTCTGAGGAGGCA
β-actin-L		AGACATCAGGGTGTGATGG
β-actin-R		TCAGGGGCTACTCTCAGCTC
NiV-F_ΔC138_-L	For plasmid construction	AGTCCAGTGTGGTGGAATTCGCCACCATGGCCGTGATC
NiV-F_ΔC138_-R		TAAACGGGCCCTCTAGATTAAGTGTTTCTCTTCTTCTCCACAATAATG
NiV-G_ΔC99_-L		GTGTGGTGGAATTCGCCACCATGCCCACTGAGTCCAAGAAGG
NiV-G_ΔC99_-R		TAAACGGGCCCTCTAGATTACTTCAGCAGGAAGCAGTTTGTG

### Production of pseudoviruses

A recombinant VSV pseudotyping system was employed to generate a NiV pseudovirus. The VSV-ΔG–GFP/NLuc pseudovirus, which carries GFP and NLuc expression cassettes in place of the VSV G glycoprotein gene, was obtained from Rong Zhang (Fudan University). To amplify the VSV-ΔG skeleton, HEK293T cells were transfected with pMD2.G (20 μg per 10-cm dish, Addgene, plasmid 12259) using Polyethylenimine Linear (PEI, Polysciences, USA). After 24 h, VSV-ΔG-GFP/NLuc pseudovirus was added at a multiplicity of infection (MOI) of 2 and incubated for 2 h. The medium was then replaced with fresh DMEM supplemented with 2% FBS. At 24 h post-infection, NiV pseudovirus-containing culture supernatants were harvested, filtered (0.45-μm pore size, Sangon, China), and stored at –80°C until use.

Pseudovirus packaging conditions were optimized across three parameters: (1) transfection reagents: PEI (Polysciences, USA), GeticoFect 2000 (Getico, China, Lipofectamine 3000 (Thermofisher, USA), GP-transfect-Mate (GenePharma, China), and Hieff Trans Universal (Yeasen, China); (2) F:G plasmid ratio (2:1, 1:1, 1:2, 1:4, 1:8); and (3) MOI (0.5, 1, 2, 4, 6). In the optimized condition, pCAGGS-NiV-M-F and pCAGGS-NiV-M-G were mixed at a 1:2 ratio (24 μg total per 10-cm dish) and transfected into HEK293T cells (2.5 × 10^7^ cells per dish) using 48 μL PEI. After 24 h, VSV-ΔG–GFP/NLuc particles were added at an MOI of 4 and incubated for 2 h. Untransfected cells served as negative controls. Cells were washed five times with PBS, and fresh DMEM containing 2% FBS was added. At 48 h post-transfection, supernatants containing NiV pseudoviruses were harvested, filtered, and concentrated using Amicon regenerated cellulose membranes (MWCO 100 kDa; Millipore, USA) by centrifugation at 4000 r/min for 10 min. Aliquots were stored at −80°C and used only once to avoid variability due to freeze-thaw cycles.

### NiV pseudovirus titration with nanoluciferase detection assay

Each batch was determined using a single-use aliquot from the pseudovirus bank. For the titration of NiV pseudovirus, a 10-fold initial dilution was made in hexaplicate wells of 96-well culture plates, followed by serial 10-fold dilutions. The last column served as the cell control. After 24 h, the culture supernatant was aspirated gently, and the NLuc activity was detected using the Nano-Glo^®^ Luciferase Assay (Promega, USA) following the manufacturer’s instructions. In brief, 50 μL of luciferase substrate/lysis buffer mixture was added to each well. After incubation at room temperature for 5 min, all the lysate was transferred to white solid 96-well plates for the detection of luminescence using a microplate luminometer (Biotek, USA). After subtracting the relative light unit (RLU) of the cell-only control group (serving as background) from the RLU of each test well, a positive well was determined as one with RLU values ten-fold higher than the cell background. The 50% tissue culture infectious dose (TCID_50_) was calculated using the Reed-Muench method.

### Flow cytometry analysis

Cells were seeded in 6-well cell culture plates overnight to form a monolayer of adherent cells, and then infected with pseudovirus. The virus-infected cells were harvested at 24 h post-infection by centrifugation at 3000 r/min for 5 min. The cells were then rinsed once with PBS and resuspended in 300 μL PBS. The preparations were analyzed by flow cytometry using a flow cytometer (Beckman, USA) equipped with FlowJo software.

### Negative staining transmission electron microscopy

Viral concentrate suspensions (20 μL) were adsorbed onto a copper grid with carbon support film (200 mesh, Beijing Zxbr Technology Co., Ltd., China) for 4 min at ambient temperature. Excess liquid was gently blotted using filter paper. Grids were then negatively stained with 2% phosphotungstic acid (Servicebio, China) for 1−2 min, followed by blotting and air-drying. Samples were examined under a transmission electron microscope (TEM, Hitachi, Japan) operated at 80 kV. Micrographs were acquired at 150,000× magnification.

### Quantitative PCR

Different cell samples’ total RNA was extracted and quantified (100 ng RNA of each sample); reverse transcription was performed with TransScript^®^ Uni All-in-One First-Strand cDNA Synthesis SuperMix for qPCR (One-Step gDNA Removal) (TransGen, China) following the manufacturer’s instructions. The quantitative PCR (qPCR) was performed in three technical replicates with *PerfectStart*^®^ Universal Green qPCR SuperMix (TransGen, China). The expressions of genes were normalized using *GAPDH*. Relative expression levels were calculated using 2^−ΔΔCt^. All primer sequences are provided in Table 1.

### Western blotting

Cells were obtained and lysed in 2× protein loading buffer (20 mmol/L Tris-HCl, 2% SDS, 100 mmol/L dithiothreitol, 20% glycerol, and 0.016% bromophenol blue). The lysates were denatured at 100℃ and the proteins were resolved by 12% SDS-PAGE. The proteins were transferred to a nitrocellulose membrane (Whatman, UK). Each membrane was blocked in 5% skim milk for 2 h at room temperature and then incubated with primary antibodies overnight at 4°C, followed by incubation with secondary antibodies for 2 h at room temperature. The protein bands were visualized using the Tanon 4600 Chemiluminescent Imaging System (BioTanon, China). Antibody against β-actin (AC006) was purchased from ABclonal (China); antibody against EFNB2 (YN0096) was purchased from Immunoway (China).

### Transfection of plasmids or siRNAs

HEK293T cells were transfected with siRNAs, or CHO-K1 and DF-1 cells were transfected with plasmids according to the manufacturer’s instructions. Briefly, 5 μL siRNAs and 10 μL GP-transfect-Mate, or 4 μg pCMV-vector/EFNB2 and 8 μL GP-transfect-Mate were incubated in 200 μL Opti-MEM (Gibco, USA) for 5 min, then mixed and incubated at room temperature for 15 min, allowing the formation of lipid−plasmid complexes. Finally, the complexes were added to 6-well tissue culture plates. After 4 h of incubation at 37°C, the medium was replaced with fresh DMEM supplemented with 2% FBS and incubated for an additional 44 h (siRNA transfection) or 20 h (plasmid transfection) before pseudovirus infection. The siRNAs targeting endogenous EFNB2 (si-EFNB2-1: 5’-AAUUCUUGAAACUUGAUGG-3’; si-EFNB2-2: 5’-UAUCUGUGGGUAUAGUACC-3’) were purchased from Sangon (China); the pCMV-vector and pCMV-EFNB2 were obtained from Shilei Zhang (Lanzhou Veterinary Research Institute).

### Pseudovirus neutralization assay

Neutralizing monoclonal antibodies 5B3, 1F5, 1H1 (targeting NiV F), and m102.4 (targeting NiV G) were produced from previously reported sequences (Byrne *et al.* 2023; Dang *et al.* 2019, 2021; Tit-Oon *et al.* 2020). Briefly, the heavy- and light-chain coding sequences for each antibody were individually cloned into pTT5 expression vectors and co-transfected into suspension CHO-K1 cells. Culture supernatants were harvested, and antibodies were purified by Protein A affinity chromatography, buffer-exchanged into PBS, concentrated, filtered, and stored frozen.

Neutralization was quantified by the reduction of luciferase signal. HEK293 cells were seeded to confluence in 96-well plates. Antibodies were fourfold serially diluted starting at 100 μg/mL, then incubated with pseudovirus at a 1:1 volume ratio for 1 h at 37°C. Note that the final antibody concentration during incubation was half of the pre-mix value (*e*.*g*., 100 μg/mL pre-mix becomes 50 μg/mL final). The antibody-pseudovirus mixtures were applied to HEK293 cells and incubated for 2 h, after which the medium was replaced with fresh DMEM containing 2% FBS. After a further 24 h, RLU was measured using the Nano-Glo Luciferase Assay Kit. Percent inhibition was calculated as the reduction in RLU relative to the control (cells plus pseudovirus without antibody). Dose-response curves were fitted using a four-parameter logistic model, and *IC*_50_ values were derived with GraphPad Prism.

### Statistical analysis

All data are presented as mean ± standard deviation (S.D.) from at least three independent experiments, using GraphPad Prism software. Significance was determined with the two-tailed independent Student’s *t*-test (*P* < 0.05) between two groups. A one-way ANOVA was followed by Tukey’s to compare multiple groups (>2).
